# Effects of nano-*Rhodiola rosea* combined with treadmill exercise on anti-exercise fatigue in rats

**DOI:** 10.3389/fnut.2024.1446944

**Published:** 2024-09-04

**Authors:** Jibing Wang, Guoyan Zhang, Duona Wang, Yuanyuan Yan, Qin Yang

**Affiliations:** International College of Football, Tongji University, Shanghai, China

**Keywords:** *Rhodiola rosea*, nano, anti-exercise fatigue, free radical, antioxidant enzyme

## Abstract

**Objective:**

To explore the potential strategies and mechanisms for enhancing the bioavailability of *Rhodiola rosea*.

**Methods:**

36 Sprague–Dawley rats (8-weeks-old) were randomly assigned to six groups (*n* = 6 per group). Groups I and II received nano-dose forms of *R. rosea*, groups III and IV received normal dose form of *R. rosea*, and groups V and VI served as distilled water control groups. Groups II, IV, and VI were combined with moderate -intensity treadmill exercise. Each group received a daily gavage with 0.5 mL of nano -*R. rosea* solution (0.01 mg/mL), normal *R. rosea* solution, and distilled water. All rats were subjected to exhaustive swimming after 4 weeks. Outcome measures include GSH-px activity, T-AOC activity, MDA content, hepatic glycogen content, and T-SOD activity.

**Results:**

For plasma MDA content, group I was lower than group III (*p* < 0.01) and group V (*p* < 0.01), group II was lower than group III (*p* < 0.01), group VI was higher than group II (*p* < 0.05) and group IV (*p* < 0.05). For plasma T-AOC activity, group II was higher than group VI (*p* < 0.01). For plasma GSH-px activity, group I was lower than group IV (*p* < 0.05), groups II, III, and IV were higher than group V (*p* < 0.05), and group V was lower than that of group VI (*p* < 0.05). For T-SOD activity of quadriceps muscle, groups I and III were higher than that in group V (*p* < 0.05).

**Conclusion:**

*R. rosea* has a positive effect on anti-exercise fatigue in rats, with the nano-dosage form of *R. rosea* showing more significant efficacy than the normal form especially combined with aerobic exercise.

## 1 Introduction

Sports nutrition supplements play an important role in relieving exercise-induced fatigue, and traditional Chinese medicines (*Panax ginseng*, *Lycium Chinense* Miller, *acanthopanax*, *astragalus*, *Panax notoginseng*, *Carthamus tinctorius*, and *Cordyceps sinensis*, etc.) have been considered to be an excellent material for the research and development of nutritional supplements for athletes (1–3). *Rhodiola rosea* is a perennial herb or subshrub in the genus Sedum (4, 5), which has anti-fatigue, anti-aging, anti-oxidation, anti-tumor and other bioactive functions (4–9). It also has a certain protective effect on the cardiovascular system (4, 10). *R. rosea* is a potent antioxidant, exerting a positive effect on anti-exercise fatigue and prolonging the duration of exhaustive exercise (11–14) through multiple mechanisms including energy reserves (15, 16) metabolites (17–21) free radicals (22–24) and antioxidants (12, 16, 20, 25–27). Importantly, *R. rosea* contains no ingredients prohibited by the International Olympic Committee (IOC) and exhibits no side effects in long-term clinical studies. Thus, it fully meets the requirements as a sports nutrition supplement (28).

Most of the studies have used the normal dosage form of *R. rosea* in studies for exercise-induced fatigue (29–31). Some scholars have also proposed to make *R. rosea* into nano dosage form so as to improve its bioavailability and enhance the effect of anti-exercise fatigue (30, 32, 33). Theoretically, the nano-dosage form of *R. rosea* can significantly increase the surface area of the particles, enhance the adherence performance, and prolong the retention time at the site of absorption. Moreover, cell walls are entirely disrupted, increasing the content of active ingredients and further stimulating previously concealed ones. So the nano-dosage form of *R. rosea* is conducive to improving the bioavailability and maximum biological activity, but there is limited research on the anti-exercise fatigue of the nano-dosage form of *R. rosea*.

Accordingly, this study focused on exploring the effects of nano-dosage and normal dosage forms of *R. rosea* combined with treadmill exercise on exercise-induced fatigue in rats, searching for possible pathways and specific mechanisms of action to enhance the anti-exercise fatigue of *R. rosea*, and providing a basis for the further development of *R. rosea* as a sports nutrition supplement.

## 2 Materials and methods

### 2.1 Preparation of *Rhodiola rosea*

*R. rosea*, produced in Tibet, was purchased by Beijing Tong Ren Tang and then made into nano-dosage and normal dosage forms of *R. rosea* by Qinhuangdao Taiji Huan Nano-Products Co, Ltd. The nano-dosage form of *R. rosea* was processed using a high-energy nano-impact grinding device in a closed physical milling system. Through rapid multidirectional motion of the grinding chamber, the material was pulverized to the nanoscale, with an average particle size of 260–280 nm. During the formal experiments, 0.01 mg/mL of *R. rosea* in nano-form and normal form were dispensed daily.

### 2.2 Animals

SpF-grade male SD rats, 8 weeks old, 36 animals, average weight 345.0 ± 29.7 g, were provided by Shanghai Sipulpikai Laboratory Animal Co. Ltd. During the whole experiment, the animals were housed in the Laboratory Animal Center of Tongji University. The temperature in the laboratory was maintained at 22 ± 2°C, the relative humidity was about 55%, and the light time was from 7:00 to 19:00. The rats were acclimatized for one week after entering the laboratory. During this period, all experimental rats were routinely fed basal feed (provided by Jiangsu Synergistic Pharmaceutical and Biological Engineering Co. Ltd.) and distilled water and were fed *ad libitum*. After the experiment, the animals were euthanized by cervical dislocation. The personnel involved in the operation, surgery, and euthanasia of animals have been trained internally by the Laboratory Animal Center of Tongji University.

### 2.3 Group

The rats were randomly divided into 6 groups, with 6 rats in each group, and the specific groupings are shown in Table 1.

**Table 1:** Rats in each group.

Groups	Information for each group	
	Supplementation	Treadmill exercise
Group I	Nano *R. rosea*	
Group II	Nano *R. rosea*	+
Group III	Normal *R. rosea*	
Group IV	Normal *R. rosea*	+
Group V	Distilled water	
Group VI	Distilled water	+

### 2.4 Procedure

After the end of adaptive feeding, the experiment was conducted on an adaptive treadmill. Throughout this adaptive phase, group II, group IV, and group VI were acclimatized to treadmill training for one week. The intensity of exercise was set at a platform inclination angle of 5°, a speed of 15 m/min, a duration of 45 min, an exercise frequency of 3 days/week, and an interval of 1 day between two consecutive exercise sessions. Concurrently, the rest of the groups were reared normally without any interventions. After this week, the rats were basically adapted to the treadmill exercise, and there was no obvious adverse reaction.

During the formal experiment, rats in each group were free to ingest food and water every day. Every morning, from 9:00 a.m. to 10:00 a.m., each group was gavaged with 0.5 mL of nano-dose *R. rosea* solution, normal-dose *R. rosea* solution, and distilled water, respectively, once a day for 4 weeks. The daily dose of nano and normal forms of *R. rosea* supplemented in groups I, II, III, and IV was 0.005 mg.

On the 29th day, all rats underwent an acclimatization swim lasting 15 min in a swimming pool maintained at a constant temperature. The size of the swimming box was 60*60*60 cm, with a water depth of 40 cm and a temperature of 28°C. On day 30, all rats were given a day of rest.

On the 31st day, each rat was subjected to an exhaustive swimming exercise wherein a load equivalent to 2% of its body weight was applied to its tail. Each group, comprising six rats, constituted one round of exhaustive swimming exercise, and the water was changed after every two rounds. The exhaustion criterion was defined as the rats’ nostrils remaining under the water surface for more than 6 s. The duration of exhaustive swimming was recorded for all rats. After exhaustion, the rats were dried with a hair dryer and returned to the cage.

On day 32, all rats were given a day of rest, and at 7:00 p.m. All rats were removed from all fodder except water. In the morning of day 33, all rats were anesthetized with 2% pentobarbital sodium at a dose of 0.025 mL/g.

**Figure 1:**
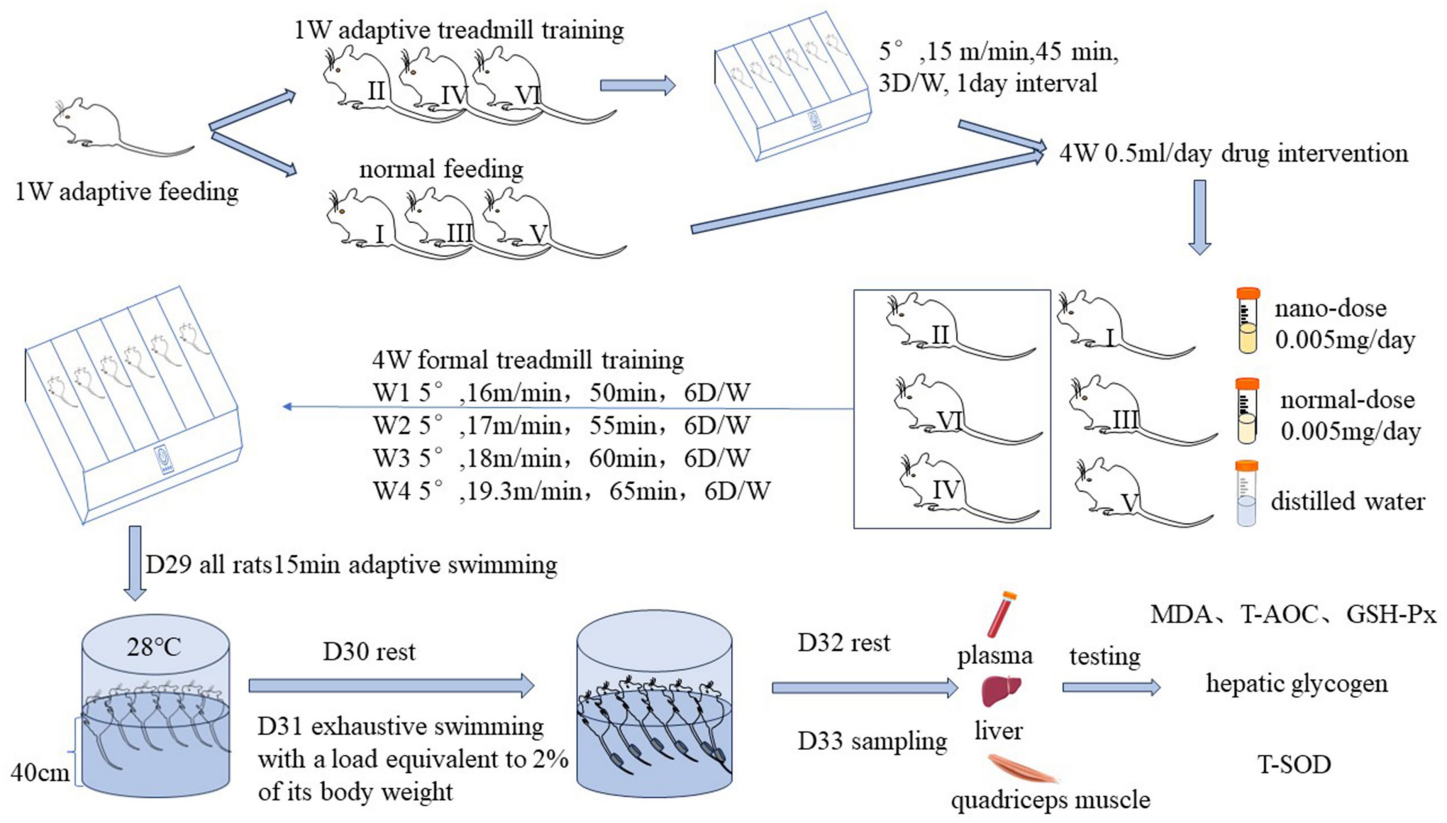
Experimental flow.

Blood samples were taken from the abdominal aorta and placed in an ethylenediaminetetraacetic acid (EDTA) anticoagulant tube. Subsequently, the samples were centrifuged for 10 min at 3,000 rpm, and the upper layer of plasma was extracted. The liver and quadriceps femoris were harvested, rinsed with saline, blotted with filter paper, wrapped in foil, and labeled with a marker pen. All samples were then stored at −80°C for subsequent testing. Figure 1 depicts the whole procedure and exercise program below.

### 2.5 Aerobic exercise

The training program for moderate-intensity treadmill exercise was modified based on the classical exercise load model established by Bedford (34). This exercise intervention was implemented specifically for groups II, IV, and VI (Table 2). The training time was daily, from 1:00 p.m. to 5:00 p.m.

**Table 2:** Exercise schedule for moderate load intensity running in rats.

Timing	Training programs			
	Running platform inclination	Speed (m/min)	Duration (minutes/times)	Frequency (days/week)
Week 1	5°	16	50	6
Week 2	5°	17	55	6
Week 3	5°	18	60	6
Week 4	5°	19.3	65	6

To guide the rats during exercise, sound stimulation and small sticks were used to stimulate their tails, prompting them to remain in the front half of the runway. When necessary, a controlled amount of electrical stimulation was applied to ensure the smooth execution of the moderate-intensity running exercise.

### 2.6 Detection of biochemical indicators

The experimental samples included plasma, liver, and quadriceps muscle. Plasma served as the sample for detecting MDA content, T-AOC activity, and GSH-px activity. The liver was used as the sample to detect hepatic glycogen content, and quadriceps muscle was used as the sample to detect total superoxide dismutase (T-SOD) activity. All kits were purchased from the Nanjing Jianjian Bioengineering Institute. The test procedures were strictly in accordance with the instructions on the kits.

The test methods are as follows: hepatic glycogen content was tested using the anthrone method (35); plasma MDA was tested using the flow-credited barbiturate (TBA, Thiobarbituric AIC) method (36); plasma T-AOC activity was measured using the phenanthroline method (37); plasma GSH-px activity was determined by the direct method of dithio-bis-nitrobenzoic acid (DINB) (38); quadriceps T-SOD activity was tested by the xanthine oxidase method (39).

### 2.7 Data analysis

All results are presented as mean ± standard deviation (X ± S), with independent samples t-test between two groups and ANOVA between multiple groups, with *p* < 0.05 as a significant difference and *p* < 0.01 as a highly significant difference. Statistical analyses were performed using GraphPad Prism 9.5 and SPSS 18.0 software.

## 3 Results

### 3.1 The duration exhaustive swimming exercise

**Figure 2:**
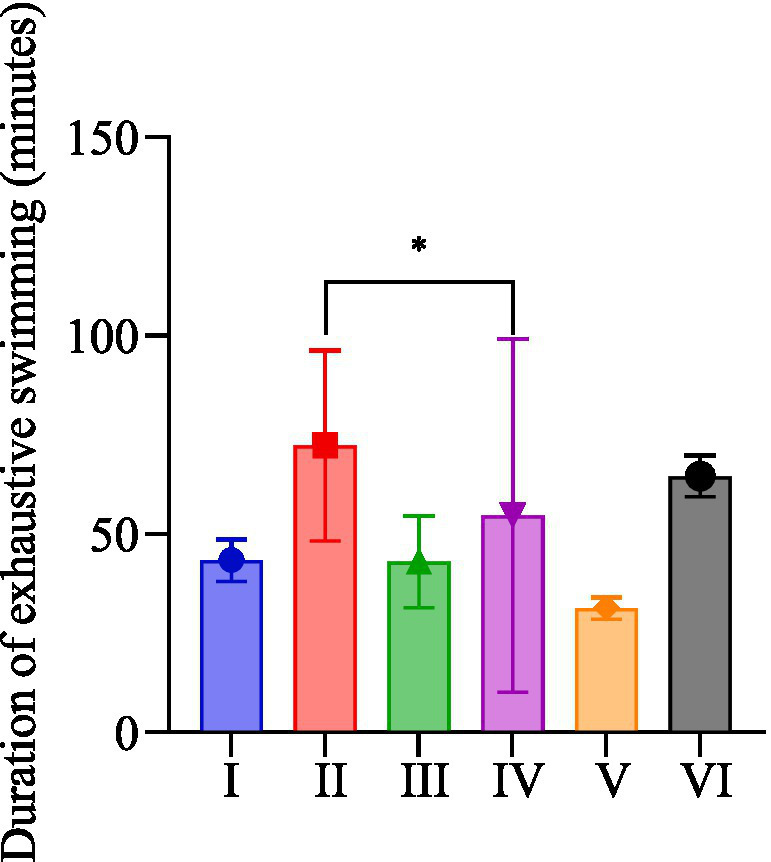
Duration of exhaustive swimming in rats of each group.

As shown in Figure 2, the duration of exhaustive swimming of rats in group II was significantly longer than that in group V (*p* < 0.05). Ranking the groups based on the duration of exhaustive swimming resulted in the following order: groups II, VI, IV, I, III, and V.

### 3.2 Hepatic glycogen content

**Figure 3:**
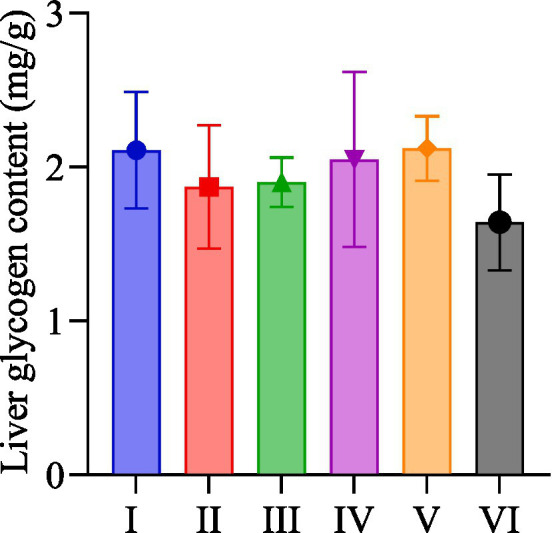
Hepatic glycogen content of rats in each group.

As shown in Figure 3, group V had the highest hepatic glycogen content, followed by group I, and group VI was the lowest. There was no significant difference between the groups.

### 3.3 MDA content, T-AOC activity, GSH-px activity of plasma

**Figure 4:**
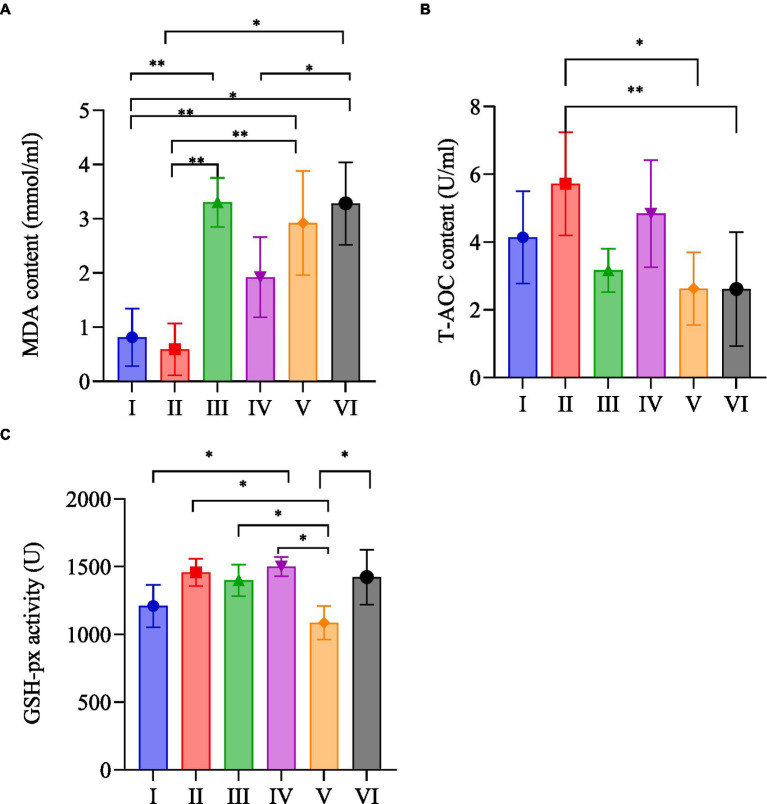
MDA content **(A)**, T-AOC activity **(B)**, GSH-px activity **(C)** of plasma in each group.

As shown in Figure 4, the plasma MDA content (Figure 4A) of rats in group II was the lowest; group I was significantly lower than group III (*p* < 0.01); group I was significantly lower than group V (*p* < 0.01); group I was significantly lower than group VI (*p* < 0.05); group II was significantly lower than group III (*p* < 0.01); group II was significantly lower than group V (*p* < 0.01); group II was significantly lower than group VI (*p* < 0.05). The plasma MDA content was ranked as group III, group VI, group V, group IV, group I, and group II.

The plasma T-AOC activity (Figure 4B) of rats in group II was significantly higher than that of group V (*p* < 0.05); group II was significantly higher than group VI (*p* < 0.01); group III was higher than group V and lower than group I. The rats of each group were ranked according to their plasma T-AOC activity as group II, group IV, group I, group III, group V, and group VI.

Plasma GSH-px activity (Figure 4C) of rats in group IV was the highest, followed by group II, and group V was the lowest. The plasma GSH-px activity of rats in group I was significantly lower than that in group IV (*p* < 0.05); group II was significantly higher than group V (*p* < 0.05); group III and group IV were significantly higher than group V (*p < 0*.05); and group V was significantly lower than group VI (*p* < 0.05).

### 3.4 T-SOD activity in quadriceps femoris muscle

**Figure 5:**
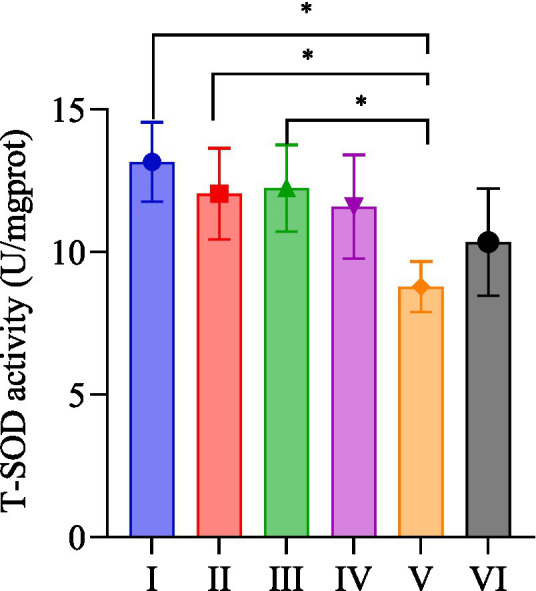
T-SOD activity of quadriceps femoris muscle in each group.

As shown in Figure 5, group I had the highest T-SOD activity, followed by group III, and group V had the lowest. The T-SOD activity of quadriceps in group I was significantly higher than that of group V (*p* < 0.05); group II was significantly higher than group V (*p* < 0.05); group III was significantly higher than group V (*p* < 0.05); and group III was significantly higher than group V (*p* < 0.05).

## 4 Discussion

The duration of exhaustive swimming is an important indicator for evaluating the degree of aerobic endurance (40). The results of the study were statistically significant only for nano-formulated *R. rosea* combined with running exercise compared with the distilled water group, which is not consistent with the results of the particles smaller than 50 nm used in the related study (32). The particle size may affect the release, absorption, and utilization of active ingredients in *R. rosea* by the organism. Furthermore, the individual variations among rats may have contributed to substantial result variation caused by exercise or *R. rosea* consumption, which could have influenced the experimental outcomes. Additionally, the rats with exercise intervention performed better in exhaustive swimming, suggesting that moderate-intensity treadmill exercise intervention can promote the anti-exercise fatigue effect of *R. rosea*, especially the nano dosage form of *R. rosea*. The nano dose is potentially more conducive to enhancing the efficacy of active ingredients in *R. rosea* within the organism compared to ordinary particle diameters, particularly in an exercise environment.

Hepatic glycogen content after exhaustive swimming exercise is an important indicator of the body’s energy recovery, which reflects the level of energy supply during exercise and its ability to resist exercise fatigue (41). In this study, the hepatic glycogen content of rats was all at a lower level. After exhaustive exercise, the hepatic glycogen has been depleted, resulting in a reduction in the original reserve. Additionally, the short rest time and inadequate food supply affect the rate of hepatic glycogen recovery in the organism. The rats in the *R. rosea* group exercised for a longer time and consumed more hepatic glycogen. After the same resting time, the content of hepatic glycogen in the R rosea group was slightly higher than that in the distilled water group. Therefore, supplementation with *R. rosea* may be more conducive to promoting the recovery of hepatic glycogen after exhaustive exercise. At a similar exhaustive and resting time, the hepatic glycogen content was higher in the nano dosage form of *R. rosea* group than in the normal dosage group. So the nano dosage form of *R. rosea* may be more favorable to promote the recovery of hepatic glycogen in the organism, which was more obvious after combined with the moderate-intensity of the treadmill exercise.

MDA is recognized as a sensitive indicator of the metabolism of free radicals, reflecting the level of lipid peroxidation and the degree of cellular damage (42). Exhaustive exercise can result in the substantial generation of reactive oxygen species by the mitochondria, leading to a significant increase in MDA content (43). Compared with the control group, supplementation with *R. rosea* demonstrated a notable impact on enhancing the elimination of MDA following exhaustive exercise. Moreover, the use of the nano dosage form of *R. rosea* exhibited more significant efficacy in reducing excess MDA than the normal dosage form after exhaustive exercise. Meanwhile, the level of plasma MDA content for nano dosage form of *R. rosea* combined with moderate-intensity treadmill exercise is higher than that of the normal dosage form of *R. rosea* combined with the same exercise regimen. This implies that nano dosage form of *R. rosea* may be more effective in mitigating the extent of lipid peroxidation following exhaustive exercise and heightening the capacity to shield the body from damage caused by free radicals.

The cellular metabolic process is always accompanied by the generation of free radicals. A large number of free radicals are generated under the state of exhaustive exercise, and the organism defends itself through the internal antioxidant defense system. Antioxidant enzymes are an important part of the antioxidant defense system, especially GSH-px and SOD. T-AOC is an important indicator for evaluating the antioxidant capacity of biologically active substances, reflecting the overall antioxidant capacity of various antioxidant macromolecules, small molecules, and enzymes in the system. The activity of GSH-px reflects the level of selenium in the organism, and it also specifically catalyzes the reduction reaction of GSH to hydrogen peroxide, which serves to protect the structural and functional integrity of cell membranes (44).

Supplementation with *R. rosea* significantly enhanced the activities of plasma T-AOC and GSH-px after exhaustive exercise, suggesting that *R. rosea* can effectively improve the level of anti-lipid peroxidation in the organism. The nano dosage form was more obvious to enhance T-AOC activity after exhaustive exercise, especially when combined with treadmill intervention. It was consistent with the changes in plasma MDA content after exhaustive exercise in the corresponding groups of rats, probably due to the fact that the small diameter of nano dosage form of *R. rosea* particles was more conducive to the release of active ingredients in *R. rosea* and the process of absorption and utilization by the organism. However, the plasma GSH-px content showed the opposite result, and the normal dosage form of *R. rosea* was more able to stimulate the activity of plasma GSH-px, which may be caused by the overall antioxidant capacity of T-AOC. And MDA focuses on reflecting the body’s overall level of lipid peroxidation and the degree of cellular damage, whereas the main target of GSH-px is hydrogen peroxide, which only represents a certain aspect of the body’s antioxidant capacity. Meanwhile, the activity of plasma GSH-px increased after the two dosage forms of *R. rosea* combined with moderate-intensity treadmill exercise. Group IV only increased by 7.2%, while group II increased by 20%. This discrepancy suggests that moderate-intensity running exercise can enhance the impact of *R. rosea*, particularly nano dosage form of *R. rosea*, on the activity of GSH-px after exhaustive exercise in rats.

SOD functions as a natural antagonist to oxygen free radicals, scavenging the superoxide anion (O^2−^). This role is integral to maintaining a balanced oxidative and antioxidant milieu in the body, thereby shielding cells from damage (45). The results showed that *R. rosea* significantly increased the T-SOD activity of the quadriceps muscle in the organism after exhaustive exercise in rats. Unlike other indexes (e.g., MDA, T-AOC, and GSH-px), the T-SOD activity in the quadriceps femoris muscle did not exhibit a clear upward trajectory and, in fact, demonstrated a slight decrease when two dosage forms of *R. rosea* were combined with moderate-intensity platform running exercise. It is conjectured that the mechanism of action on T-SOD activity in quadriceps muscle may be through one or more SOD fractions. The combination of moderate-intensity treadmill running exercise with *R. rosea* may not have achieved the expected benign cycle, as the moderate-intensity running exercise may have disrupted the signaling pathway associated with a specific fraction of SOD activity targeted by *R. rosea*. This interference likely led to disturbances in the pathway, contributing to an overall decline in the T-SOD activity of quadriceps. Alternatively, the mechanism may be diametrically opposed, wherein medium-intensity running exercise diminishes the fraction of SOD activity that *R. rosea* can enhance, and reciprocally, *R. rosea* reduces the portion of SOD activity that can be augmented by medium-intensity running exercise. The mutual weakening effect between the two decreased the overall T-SOD activity of the quadriceps muscle. To validate and elucidate this potential interaction, further studies should be undertaken.

In summary, nano-dose showed a stronger anti-fatigue effect compared with normal-dose, and the underlying mechanism may involve multiple molecular pathways. The application of nanotechnology may have enhanced the solubility and bioavailability of the active ingredients in *Rhodiola rosea*, enabling controlled release and thus promoting more efficient absorption and distribution (46, 47). Active ingredients within the nano-*R. rosea*, such as salidroside and rosavin, may modulate intracellular signaling pathways like AMPK and mTOR (48), enhancing cellular energy metabolism and antioxidant capacity, thereby improving anti-fatigue capabilities. In addition, nano-*rhodiola rosea* may regulate mitochondrial function (49), improve the activity of antioxidant enzymes in cells, reduce the generation and accumulation of free radicals, and thus reduce the oxidative stress damage caused by exercise.

Other anti-fatigue drugs have also shown significant results in improving athletic performance and reducing fatigue. Chen et al. (50), evaluated the potential beneficial effect of nano-bubble curcumin extract in reducing exercise-related injuries and improving performance. Results indicated that nano-bubble curcumin extract supplementation alters the gut microbiota composition and aids in overcoming physical fatigue. Liu et al. (51) and Yun et al. (52), examined the synergistic effects of combining *R. rosea* and caffeine supplementation on muscle endurance and explosiveness in SD rats and human subjects. In both the rat model and human subjects, the *R. rosea* + caffeine group demonstrated significantly greater physical performances compared to the use of *R. rosea* or caffeine supplements individually. When used alone, *R. rosea* as an adaptogen herb may have a milder and longer-lasting anti-fatigue effect. Caffeine is a central nervous system stimulant that can temporarily increase alertness and physical performance, but its long-term use can lead to dependence and side effects such as heart palpitations and insomnia.

Although *Rhodiola rosea* is considered safe for traditional use, the application of nanotechnology may present new safety concerns. The high surface area and surface activity of nanoparticles may increase their bioreactivity *in vivo*, leading to potential toxic reactions. For example, nano *R. rosea* may trigger cell damage or inflammatory responses by affecting the integrity and function of cell membranes. In addition, nanoparticles may accumulate in the body, and long-term accumulation may have negative effects on organs such as the liver and kidneys (53). Further toxicological studies are needed to assess its safety.

## 5 Highlights and limitations

This study innovatively combines nano-Chinese medicine technology with exercise intervention to conduct an in-depth investigation into the anti-fatigue effects of *Rhodiola rosea*. It reveals the significant advantages of nano-dosage of *R. rosea* over the normal formula in terms of enhancing bioavailability and antioxidant capacity. A comprehensive assessment of its efficacy was made through a series of biochemical indicators, providing a new perspective for the modern application of traditional Chinese medicine. The advent of nanotechnology has introduced a novel approach to the processing of traditional Chinese medicine, with the physical nanotechnology used in this study also offering a new attempt for further processing of other Chinese medicine-based sports nutrition supplements.

However, there are limitations, including insufficient exploration of dose effects, long-term effects, and individual differences. No detailed analysis was performed of changes in body weight in rats, which may affect the accuracy of drug dosage and metabolic rate. In addition, the potential side effects of *Rhodiola rosea* have been poorly discussed, and direct human research validation is lacking. Although nanotechnology has been applied to improve bioavailability, the safety and long-term accumulation effects of nanoparticles still require further toxicological studies. Future research could consider the use of nano-biotechnology, such as improving the targeting of traditional Chinese medicine effects through nano-drug delivery technology. This could more specifically reveal the mechanism of resistance to exercise fatigue of *R. rosea* and could also be more conducive to enhancing the efficacy of *Rhodiola rosea* in combating exercise fatigue.

## 6 Conclusion

*R. rosea* has a positive effect on anti-exercise fatigue in rats. Notably, the anti-fatigue effect of nano-formulations is more significant than that of normal formulations when combined with aerobic exercise. The probable mechanism is to reduce the generation of free radicals or scavenging of free radicals by regulating the oxidative stress system, which is stronger than that of the common formulations.

## Data Availability

The original contributions presented in the study are included in the article/supplementary material, further inquiries can be directed to the corresponding author.
